# Interim Effectiveness Estimates of 2024 Southern Hemisphere Influenza Vaccines in Preventing Influenza-Associated Hospitalization — REVELAC-i Network, Five South American Countries, March–July 2024

**DOI:** 10.15585/mmwr.mm7339a1

**Published:** 2024-10-03

**Authors:** Erica E. Zeno, Francisco Nogareda, Annette Regan, Paula Couto, Marc Rondy, Jorge Jara, Carla Voto, Maria Paz Rojas Mena, Nathalia Katz, Maria del Valle Juarez, Estefanía Benedetti, Francisco José de Paula Júnior, Walquiria Aparecida Ferreira da Almeida, Carlos Edson Hott, Paula Rodríguez Ferrari, Natalia Vergara Mallegas, Marcela Avendaño Vigueras, Chavely Domínguez, Marta von Horoch, Cynthia Vazquez, Eduardo Silvera, Hector Chiparelli, Natalia Goni, Laura Castro, Perrine Marcenac, Rebecca J. Kondor, Juliana Leite, Martha Velandia, Eduardo Azziz-Baumgartner, Ashley L. Fowlkes, Daniel Salas, REVELAC-i Network, REVELAC-i Network, Estefania Benedetti, Andrea Pontoriero, Maria del Valle Juarez, Nathalia Katz, Maria Paz Rojas Mena, Carla Jimena Voto, Walquiria Aparecida Ferreira da Almeida, Daiana Araújo da Silva, Francisco José de Paula Júnior, Felipe Cotrim de Carvalho, Ana Catarina de Melo Araujo, Greice Madeleine Ikeda do Carmo, Carlos Edson Hott, Miriam Teresinha Furlam Prando Livorati, Marcela Avendaño, María Fernanda Olivares Barraza, Patricia Bustos, Paula Rodríguez Ferrari, Natalia Vergara Mallegas, Rodrigo Fasce Pineda, Silvia Battaglia, Marta Von Horoch, Chavely Domínguez, Maria José Ortega, Elena Penayo, Cynthia Vázquez, Hector Chiparelli, Natalia Goñi, Karina Griot, Jose Eduardo Silvera, Daiana Tritten, Steven Tapia Villacís

**Affiliations:** ^1^Influenza Division, National Center for Immunization and Respiratory Diseases, CDC; ^2^Epidemic Intelligence Service, CDC; ^3^Pan American Health Organization, Washington, DC; ^4^School of Nursing and Health Professions, University of San Francisco, San Francisco, California; ^5^Área de Vigilancia, Dirección de Epidemiología, Ministerio de Salud, Buenos Aires, Argentina; ^6^Dirección de Control de Enfermedades Inmunoprevenibles, Ministerio de Salud, Buenos Aires, Argentina; ^7^Laboratorio Nacional de Referencia INEI-ANLIS–Dr. Carlos G. Malbrán, Buenos Aires, Argentina; ^8^Ministry of Health, Brasília, Brazil; ^9^Departamento de Epidemiologia - Ministerio de Salud, Santiago, Chile; ^10^Dirección General de Vigilancia de la Salud, Ministerio de Salud Pública y Bienestar Social, Asunción, Paraguay; ^11^Programa Ampliado de Inmunizaciones, Ministerio de Salud Pública y Bienestar Social, Asunción, Paraguay; ^12^Laboratorio Central de Salud Pública, Ministerio de Salud Pública y Bienestar Social, Asunción, Paraguay; ^13^Departamento de Vigilancia en Salud, Control de Infecciones Hospitalarias, Ministerio de Salud Pública, Montevideo, Uruguay; ^14^Departamento de Laboratorios de Salud Pública, Ministerio de Salud Pública, Montevideo, Uruguay.; INEI-ANLIS–Dr. Carlos G. Malbrán, Buenos Aires, Argentina; INEI-ANLIS–Dr. Carlos G. Malbrán, Buenos Aires, Argentina; Ministry of Health, Buenos Aires, Argentina; Ministry of Health, Buenos Aires, Argentina; Ministry of Health, Buenos Aires, Argentina; Ministry of Health, Buenos Aires, Argentina; Ministry of Health, Brasília, Brazil; Ministry of Health, Brasília, Brazil; Ministry of Health, Brasília, Brazil; Ministry of Health, Brasília, Brazil; Ministry of Health, Brasília, Brazil; Ministry of Health, Brasília, Brazil; Ministry of Health, Brasília, Brazil; Ministry of Health, Brasília, Brazil; Ministry of Health, Santiago, Chile; Ministry of Health, Santiago, Chile; Ministry of Health, Santiago, Chile; Ministry of Health, Santiago, Chile; Ministry of Health, Santiago, Chile; Ministry of Health, Santiago, Chile; Ministry of Public Health and Social Welfare, Asunción, Paraguay; Ministry of Public Health and Social Welfare, Asunción, Paraguay; Ministry of Public Health and Social Welfare, Asunción, Paraguay; Ministry of Public Health and Social Welfare, Asunción, Paraguay; Ministry of Public Health and Social Welfare, Asunción, Paraguay; Ministry of Public Health and Social Welfare, Asunción, Paraguay; Ministry of Public Health, Montevideo, Uruguay; Ministry of Public Health, Montevideo, Uruguay; Ministry of Public Health, Montevideo, Uruguay; Ministry of Public Health, Montevideo, Uruguay; Ministry of Public Health, Montevideo, Uruguay; Ministry of Public Health, Montevideo, Uruguay

**Summary**

**What is already known about this topic?**

Influenza vaccine effectiveness (VE) varies by season.

**What is added by this report?**

In five South American countries (Argentina, Brazil, Chile, Paraguay, and Uruguay) the 2024 Southern Hemisphere seasonal influenza vaccine reduced the risk for influenza-associated hospitalization among high-risk groups by 35%. VE might be similar in the Northern Hemisphere if similar A(H3N2) viruses predominate during the 2024–25 influenza season.

**What are the implications for public health practice?**

CDC recommends that all eligible persons aged ≥6 months receive seasonal influenza vaccine. Early antiviral treatment can complement vaccination to protect against severe influenza-related morbidity.

## Abstract

To reduce influenza-associated morbidity and mortality, countries in South America recommend annual influenza vaccination for persons at high risk for severe influenza illness, including young children, persons with preexisting health conditions, and older adults. Interim estimates of influenza vaccine effectiveness (VE) from Southern Hemisphere countries can provide early information about the protective effects of vaccination and help guide Northern Hemisphere countries in advance of their season. Using data from a multicountry network, investigators estimated interim VE against influenza-associated severe acute respiratory illness (SARI) hospitalization using a test-negative case-control design. During March 13–July 19, 2024, Argentina, Brazil, Chile, Paraguay, and Uruguay identified 11,751 influenza-associated SARI cases; on average, 21.3% of patients were vaccinated against influenza, and the adjusted VE against hospitalization was 34.5%. The adjusted VE against the predominating subtype A(H3N2) was 36.5% and against A(H1N1)pdm09 was 37.1%. These interim VE estimates suggest that although the proportion of hospitalized patients who were vaccinated was modest, vaccination with the Southern Hemisphere influenza vaccine significantly lowered the risk for hospitalization. Northern Hemisphere countries should, therefore, anticipate the need for robust influenza vaccination campaigns and early antiviral treatment to achieve optimal protection against influenza-associated complications.

## Introduction

Influenza epidemics typically occur during the cool weather months of April–September in the Southern Hemisphere and October–May in the Northern Hemisphere. Every year, it is estimated that influenza results in 716,000–829,000 hospitalizations and 41,007–71,710 deaths throughout the Americas (*1*,*2*). To prevent influenza-associated morbidity and mortality, most countries in the Americas have implemented influenza vaccination programs (*3*). The Pan American Health Organization (PAHO) Network for the Evaluation of Vaccine Effectiveness in Latin America and the Caribbean - influenza (Red para la Evaluación de Vacunas en Latino América y el Caribe - influenza [REVELAC-i])^†^ provides timely information about the vaccination status of hospitalized influenza patients and vaccine effectiveness (VE), which guides public health messaging and influenza vaccine composition decisions for each Southern Hemisphere season. Southern Hemisphere VE estimates also herald what Northern Hemisphere jurisdictions might anticipate about VE if the same influenza viruses circulate during their upcoming influenza season.

## Methods

### Data Sources

Patients with severe acute respiratory illness (SARI), defined as an acute respiratory illness with either a history of fever or measured body temperature ≥100.4°F (≥38°C), cough, and onset ≤10 days before hospitalization, were identified through the SARInet Plus network.^§^^,^^¶^ Respiratory specimens were tested for influenza virus by reverse transcription–polymerase chain reaction (RT-PCR) and typed and subtyped in national reference laboratories.

The study population comprised SARI patients in three mutually exclusive PAHO target groups for vaccination: young children, persons with comorbidities, and older adults; definitions of young children and older adults varied among the countries.** March–July 2024 data were pooled from 2,535 hospitals, including 30 in Argentina, 2,477 in Brazil, 13 in Chile, five in Paraguay, and 10 in Uruguay. VE evaluation began 2 weeks after commencement of each country’s influenza vaccination campaign.^††^ All countries used World Health Organization (WHO)–recommended egg-based Southern Hemisphere formulations. Argentina, Brazil, Chile, and Uruguay used trivalent vaccines containing antigens from A/Victoria/4897/2022 (H1N1)pdm09–like virus, A/Thailand/8/2022 (H3N2)–like virus, and B/Austria/1359417/2021 (B/Victoria lineage)–like virus. Paraguay used quadrivalent vaccines that also contained the B/Yamagata lineage–like virus.^§§^

### Study Design

VE against influenza-associated hospitalization was estimated using a test-negative case-control study design. Case-patients were SARI patients who received a positive influenza RT-PCR test result. Control patients were SARI patients who received negative RT-PCR test results for both influenza virus and SARS-CoV-2 (*4*). Vaccination status was ascertained using unique patient identifiers to link to national electronic immunization records. SARI patients who received the 2024 influenza vaccine ≥14 days before symptom onset were considered vaccinated. Those not vaccinated before symptom onset were considered unvaccinated, and those vaccinated 0–13 days before symptom onset were excluded from the evaluation.

### Data Analysis

VE was calculated by comparing the odds of influenza vaccination between influenza test-positive SARI case-patients and influenza test-negative control patients using multivariable logistic regression, overall and by target group.^¶¶^ To reduce potential confounding, models were adjusted for country, sex, age in years (cubic spline), week of symptom onset (cubic spline), and presence of at least one comorbidity. Analyses were stratified by influenza type and subtype when at least five patients contributed to each stratum or when the width of the 95% CI was <140 percentage points from lower to upper bounds. Because Brazil accounted for the majority of SARI cases, a sensitivity analysis excluding Brazil was conducted. This activity was reviewed by CDC, deemed not research, and conducted consistent with applicable federal law and CDC policy.***

## Results

### Characteristics of the Study Population

During March 13–July 19, 2024, among a total of 111,856 SARI patients identified, 100,260 were excluded because of missing influenza RT-PCR results (70,055); ineligibility or not being in a vaccine target group (14,245); symptom onset before vaccine availability, outside the influenza season, or after hospital admission (7,581); unknown vaccination status or vaccination date (5,157); specimen collection >10 days after symptom onset (1,220); vaccination <14 days before symptom onset (911); receipt of a positive SARS-CoV-2 test result (503); not meeting the SARI case definition (251); or missing demographic information (201). A total of 11,751 patients met inclusion criteria, including 630 (5.4%) from Argentina, 9,095 (77.4%) from Brazil, 1,584 (13.5%) from Chile, 162 (1.4%) from Paraguay, and 280 (2.4%) from Uruguay (Table 1). Overall, 6,851 (58.3%) patients were young children, 1,702 (14.5%) were older children and adults with comorbidities, and 3,198 (27.2%) were older adults. The majority of SARI patients in Brazil were in the young children target group.

**TABLE 1:** Seasonal vaccination status and influenza test results among hospitalized patients with severe acute respiratory illness, by select characteristics — REVELAC-i Network, five South American countries,* March–July 2024

Characteristic	SARI patients					
	No. (column %)	Vaccinated^†^ no. (row %)	p-value^§^	Influenza test result, no. (row %)		p-value^§^
				Positive	Negative	
**Overall**	**11,751**	**2,508 (21.3)**	**—**	**3,848 (32.7)**	**7,903 (67.3)**	**—**
**Target group** ^¶^						
Young children	6,851 (58.3)	1,326 (19.4)	<0.001	1,091 (16.0)	5,741 (84.0)	<0.001
Persons with comorbidities	1,702 (14.5)	246 (14.5)		858 (50.4)	844 (49.6)	
Older adults	3,198 (27.2)	936 (29.3)		1,894 (59.2)	1,304 (40.8)	
**Sex**						
Female	5,780 (49.3)	1,246 (21.6)	0.577	2,068 (35.8)	3,709 (64.2)	<0.001
Male	5,971 (50.8)	1,262 (21.1)		1,780 (29.8)	4,180 (70.2)	
**Influenza test result**						
Negative for influenza	7,903 (67.3)	1,804 (22.8)	<0.001	—	7,903 (100.0)	—
Positive for any influenza type A or B	3,848 (32.7)	704 (18.3)	—	3,848 (100.0)	—	
Positive for influenza A	3,794 (98.6)	697 (18.4)	<0.001	3,794 (100.0)	—	
Positive for influenza A(H3N2) subtype	1,628 (42.9)	292 (17.9)	<0.001	1,628 (100.0)	—	
Positive for influenza A(H1N1)pdm09 subtype	754 (19.9)	136 (18.0)	<0.001	754 (100.0)	—	
Positive for unknown A subtype	1,412 (37.2)	269 (19.1)	—	1,412 (100.0)	—	
Positive for influenza type B	26 (0.7)	5 (19.2)	<0.001	26 (100.0)	—	
Positive for unknown influenza virus type	28 (0.7)	2 (7.1)	—	28 (100.0)	—	
**Country**						
**Argentina**	630 (5.4)	125 (19.8)	—	203 (32.2)	427 (67.8)	—
Young children	228 (36.2)					
Persons with comorbidities	254 (40.3)					
Older adults	148 (23.5)					
**Brazil**	9,095 (77.4)	1,840 (20.2)		2,945 (32.4)	6,150 (67.6)	
Young children	6,080 (66.9)					
Persons with comorbidities	896 (9.9)					
Older adults	2,119 (23.3)					
**Chile**	1,584 (13.5)	507 (32.0)		537 (34.3)	1,028 (65.7)	
Young children	350 (22.1)					
Persons with comorbidities	493 (31.5)					
Older adults	741 (47.3)					
**Paraguay**	162 (1.4)	14 (8.6)		46 (28.4)	116 (71.6)	
Young children	76 (46.9)					
Persons with comorbidities	0 (—)					
Older adults	86 (53.1)					
**Uruguay**	280 (2.4)	22 (7.9)		112 (40.0)	168 (60.0)	
Young children	117 (41.8)					
Persons with comorbidities	59 (21.1)					
Older adults	104 (37.1)					

**Abbreviations:** REVELAC-i = La Red para la Evaluación de Vacunas en Latino América y el Caribe - influenza; SARI = severe acute respiratory infection.

* Argentina, Brazil, Chile, Paraguay, and Uruguay.

^†^ Patients who received ≥1 dose of the 2024 season influenza vaccine ≥14 days before symptom onset were considered vaccinated; patients who did not receive any influenza vaccine during the 2024 season by the time of symptom onset were considered unvaccinated. Patients vaccinated 0–13 days before symptom onset or who received positive SARS-CoV-2 reverse transcription–polymerase chain reaction test results were excluded from the evaluation to avoid the risk of confounding.

^§^ A Pearson’s chi-square test was used to ascertain whether there were differences in the numbers of persons who were vaccinated and unvaccinated or who received positive and negative influenza test results.

^¶^ Target groups are included as mutually exclusive groups of patients considered to be at high risk for severe outcomes associated with influenza infection. Young children were defined as those aged 6 months–2 years (Argentina), 6 months–3 years (Paraguay), 6 months–5 years (Chile and Uruguay), and 6 months–6 years (Brazil). Older adults were defined as those aged ≥60 years (Brazil and Paraguay) and aged ≥65 years (Argentina, Chile, and Uruguay). The preexisting conditions tracked by REVELAC-i are asthma, cancer, hypertension, diabetes, cardiovascular disease, respiratory disease (excluding asthma), obesity, and immunocompromise.

### Characteristics of Influenza Case-Patients

Approximately one third (32.7%; 3,848) of SARI patients received a positive influenza test result; most (98.6%) viruses identified were influenza A viruses. Only 26 (0.7%) patients were infected with influenza B viruses, all of which were B/Victoria lineage; influenza virus type was missing for 28 (0.7%) case-patients. Among 2,382 (61.9%) influenza A viruses that were subtyped, 1,628 (68.3%) were A(H3N2) and 754 (31.7%) A(H1N1)pdm09 (Figure). The majority of influenza case-patients were older adults (59.2%), followed by persons with comorbidities (50.4%); the lowest percentage of cases (16.0%) occurred among young children (p<0.001).

**Figure:**
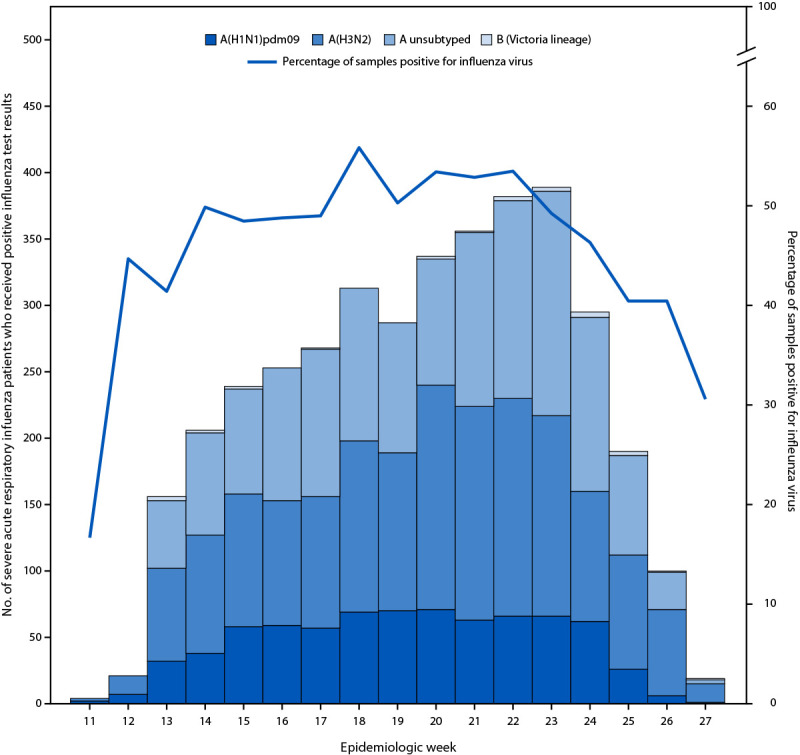
FIGURE. Patients hospitalized with severe acute respiratory infection who received positive influenza virus test results,* by epidemiologic week,^†^ (N = 11,751) — REVELAC-i Network, five South American countries,^§^ March–July 2024 * By reverse transcription–polymerase chain reaction testing at national reference laboratories. ^†^ Epidemiologic week 11 began on March 10, 2024; epidemiologic week 27 ended on July 6, 2024. ^§^ Argentina, Brazil, Chile, Paraguay, and Uruguay.

### Vaccination Status of Case- and Control Patients

Overall, 21.3% of SARI patients were vaccinated; vaccination coverage varied by target group: 29.3% of older adults, 19.4% of young children, and 14.5% of persons with comorbidities were vaccinated (p<0.001) (Table 1). Among 3,848 influenza case-patients, 704 (18.3%) had received a 2024 seasonal influenza vaccine compared with 1,804 of 7,903 (22.8%) control patients (p<0.001).

### Vaccine Effectiveness

The adjusted VE against any influenza-associated hospitalization was 34.5% overall, including 58.7% among persons with comorbidities, 39.0% among young children, and 31.2% among older adults (Table 2). Among influenza A subtypes, VE was 36.5% against the predominating A(H3N2) and 37.1% against A(H1N1)pdm09. As of July 19, too few influenza B detections were available to estimate VE. Adjusted VE against SARI from any influenza virus was 42.2% in Argentina, 30.3% in Brazil, 56.9% in Chile, and 61.0% in Uruguay; VE was not calculated for Paraguay because data were insufficient. In the sensitivity analysis excluding Brazil, the adjusted VE for all other countries was 56.5%.

**TABLE 2:** Interim 2024 Southern Hemisphere seasonal influenza vaccine effectiveness against influenza — REVELAC-i Network, five South American countries,* March–July 2024

Influenza type/Target group^¶^ and country	Influenza test-positive case-patients^†^		Influenza test-negative control patients		Vaccine effectiveness^§^	
	Total no.	Vaccinated, no. (%)	Total no.	Vaccinated, no. (%)	Unadjusted % (95% CI)	Adjusted^§^ % (95% CI)
**Any influenza type A or B**						
**Overall**	**3,848**	**704 (18.3)**	**7,889**	**1,804 (22.9)**	**24.3 (16.5 to 31.4)**	**34.5 (26.4 to 41.6)**
Young children	**1,096**	141 (12.9)	**5,741**	1,185 (20.6)	43.1 (31.0 to 53.0)	39.0 (25.6 to 50.0)
Persons with comorbidities	**858**	72 (8.4)	**844**	174 (20.6)	64.7 (52.3 to 74.1)	58.7 (43.4 to 69.8)
Older adults	**1,894**	491 (25.9)	**1,304**	445 (34.1)	32.4 (21.0 to 42.3)	31.2 (18.3 to 42.0)
**Influenza type A**						
**Overall**	**3,794**	**697 (18.4)**	**7,903**	**1,804 (22.8)**	**23.9 (16.0 to 31.1)**	**34.2 (26.0 to 41.4)**
Young children	**1,081**	140 (13.0)	**5,755**	1,185 (20.6)	42.6 (30.4 to 52.7)	38.1 (24.4 to 49.2)
Persons with comorbidities	**830**	70 (8.4)	**844**	174 (20.6)	64.5 (51.9 to 74.0)	58.3 (42.6 to 69.7)
Older adults	**1,883**	487 (25.9)	**1,304**	445 (34.1)	32.7 (21.2 to 42.5)	31.4 (18.5 to 42.2)
**Influenza A(H1N1)pdm09 subtype**						
**Overall**	**754**	**136 (18.0)**	**7,903**	**1,804 (22.8)**	**25.6 (9.4 to 39.1)**	**37.1 (21.9 to 49.4)**
Young children	**204**	16 (7.8)	**5,755**	1,185 (20.6)	67.4 (45.3 to 81.8)	60.0 (31.7 to 76.6)
Persons with comorbidities	**149**	12 (8.1)	**844**	174 (20.6)	66.3 (37.3 to 83.4)	57.6 (19.1 to 77.8)
Older adults	**400**	108 (27.0)	**1,304**	445 (34.1)	28.6 (7.9 to 44.9)	27.8 (5.1 to 45.0)
**Influenza A(H3N2) subtype**						
**Overall**	**1,628**	**292 (18.0)**	**7,903**	**1,804 (22.8)**	**26.1 (15.1 to 35.7)**	**36.5 (25.8 to 45.7)**
Young children	**453**	62 (13.8)	**5,755**	1,185 (20.6)	38.8 (19.2 to 54.3)	38.4 (17.3 to 54.1)
Persons with comorbidities	**384**	28 (7.3)	**844**	174 (20.6)	69.7 (53.6 to 80.8)	67.4 (49.3 to 79.0)
Older adults	**791**	202 (25.5)	**1,304**	445 (34.1)	33.8 (19.0 to 45.9)	30.8 (14.4 to 44.0)
**Influenza type B**						
**Overall**	**26**	**5 (19.2)**	**7,903**	**1,804 (22.8)**	NC**	NC**
Young children	**8**	0 (—)	**5,755**	1,185 (20.6)	NC**	NC**
Persons with comorbidities	**8**	1 (12.5)	**844**	174 (20.6)	NC**	NC**
Older adults	**10**	4 (40.0)	**1,304**	445 (34.1)	NC**	NC**
**Any influenza type A or B**						
Argentina	**203**	27 (13.3)	**427**	98 (23.0)	48.5 (16.8 to 68.9)	42.2 (6.9 to 64.1)
Brazil	**2,945**	561 (19.1)	**6,150**	1,279 (20.8)	10.4 (–0.3 to 19.9)	30.3 (19.9 to 39.4)
Chile	**542**	109 (20.3)	**1,042**	398 (38.2)	59.3 (47.7 to 68.4)	56.9 (42.5 to 67.7)
Paraguay	**46**	1 (2.2)	**116**	13 (11.2)	NC**	NC**
Uruguay	**112**	6 (5.4)	**168**	16 (9.5)	46.2 (–50.9 to 83.3)	61.0 (–11.5 to 86.4)

**Abbreviation:** NC = not calculated.

* Argentina, Brazil, Chile, Paraguay, and Uruguay.

^†^ Reverse transcription polymerase–chain reaction testing for influenza was conducted at national reference laboratories.

^§^ Vaccine effectiveness estimated from logistic regression model adjusting for participating country, sex, age in years (fit as cubic spline), week of onset of symptoms (fit as cubic spline), and presence of at least one comorbidity.

^¶^ Young children were defined as those aged 6 months–2 years (Argentina), 6 months–3 years (Paraguay), 6 months–5 years (Chile and Uruguay), and 6 months–6 years (Brazil). Older adults were defined as those aged ≥60 years (Brazil and Paraguay) and aged ≥65 years (Argentina, Chile, and Uruguay).

** Percentage was NC when fewer than five patients were in each of the categories.

### Genetic Characterization of Viruses Reported by REVELAC-i Countries

As of August 12, most A(H1N1)pdm09 viruses reported by REVELAC-i countries to the Global Initiative on Sharing All Influenza Data^†††^ were clade 5a.2a.1 (64.2%) or 5a.2a (35.8%). Most reported A(H3N2) viruses were clade 2a.3a.1 subclade J.2 (92.3%), subclade J.1 (7.5%), or subclade J (0.1%).^§§§^

## Discussion

This evaluation suggests that while only one in five SARI patients had received the 2024 influenza vaccine, those who were vaccinated were at significantly lower risk for hospitalization from any influenza virus infection, including the predominant influenza A(H3N2) and influenza A(H1N1)pdm09 subtypes. Although South American countries prioritized young children, persons with comorbidities, and older adults for vaccination to prevent influenza illness complications, the documented influenza vaccination coverage levels (21.3%) were below pre–COVID-19 norms. This finding is consistent with postpandemic declines in vaccination coverage across the Americas associated with vaccine misinformation, hesitancy, and disruptions in routine immunization services, prevalent during the COVID-19 pandemic (*3*). Vaccination remains one of the most effective measures to prevent influenza-associated complications, including death.^¶¶¶^ Annual influenza vaccination should be encouraged for young children, persons with comorbidities, and older adults (*5*). Influenza vaccine postintroduction evaluations and knowledge, attitudes, and practices surveys might identify additional reasons for low coverage and strategies for improved coverage for the next Southern Hemisphere season.

Despite the low influenza vaccination coverage, those vaccinated were protected against hospitalization. The 34.5% REVELAC-i VE against all influenza-associated hospitalization was within historical ranges of 34%–53% against A(H3N2) and 18%–56% against A(H1N1)pdm09 (*6*). Vaccination likely prevented 36.5% of influenza A(H3N2)–associated and 37.1% of influenza A(H1N1)pdm09–associated hospitalizations. VE was lowest in Brazil, likely because a higher proportion of cases in Brazil occurred among young children, a population with a VE estimate in the lower range among the three target groups. If these clades predominate during the Northern Hemisphere influenza season and the updated A/Thailand/8/2022 (H3N2)–like virus antigen provides similar protection against clade 2a.3a.1, health authorities might anticipate similar levels of protection from the 2024–25 vaccine (*7*). To enhance this year’s modest influenza vaccine protection against hospitalization, providers should treat patients with suspected or confirmed influenza as soon as possible with antivirals.

### Limitations

The findings in this report are subject to at least five limitations. First, small interim-estimate sample sizes precluded the estimation of VE against influenza B. Second, although the analyses were robust, 63% of patients were excluded because they did not receive RT-PCR results in time for the interim analysis. Third, Brazil, which is approximately three times as populous as the other countries combined,**** accounted for approximately 80% of the study population and included a higher percentage of SARI patients in the young children target group compared with that in other countries. Overall analyses were adjusted for country and target group but might still be more representative of Brazil’s VE estimate. Fourth, this analysis does not distinguish between young children who received 1 or 2 vaccine doses; VE might be higher among young children who received 2 influenza vaccine doses. Finally, these results might not be generalizable to other target groups or to countries with different viral circulation and vaccination strategies.

### Implications for Public Health Practice

Interim VE estimates from the REVELAC-i Network suggest that influenza vaccines are effective in preventing approximately one third of influenza-related hospitalizations among groups prioritized for vaccination. Although Southern Hemisphere influenza VE is not necessarily predictive of Northern Hemisphere VE, it can help the Northern Hemisphere plan contingencies for vaccination demand and use. These data suggest that influenza vaccine demand was still low post–COVID-19 but that vaccination prevented approximately one third of influenza-associated hospitalizations among groups at high risk for influenza-associated complications. These findings support CDC and WHO’s recommendation that all eligible persons aged ≥6 months should receive influenza vaccination (*5*,*8*). If similar influenza viruses continue to predominate during Northern Hemisphere influenza season and the updated A/Thailand/8/2022 (H3N2)–like antigen provides similar protection against circulating influenza A(H3N2) viruses, health authorities might anticipate similar levels of protection. Nonpharmaceutical measures, such as hand washing and mask use, and early antiviral treatment can complement vaccination for stronger protection against influenza illness and its complications.

## Footnotes

* These authors contributed equally to this report.

†
https://www.paho.org/en/network-evaluation-vaccine-effectiveness-latinamerica-and-caribbean-influenza-revelac-i

§
https://sarinet.org

¶
https://www3.paho.org/revelac-i/wp-content/uploads/2015/10/2015-cha-operational-guidelines-sentinel-sari.pdf

** Young children were defined as those aged 6 months–2 years (Argentina), 6 months–3 years (Paraguay), 6 months–5 years (Chile and Uruguay), and 6 months–6 years (Brazil). Older adults were defined as those aged ≥60 years (Brazil and Paraguay) and those aged ≥65 years (Argentina, Chile, and Uruguay). The preexisting conditions tracked by REVELAC-i are asthma, cancer, hypertension, diabetes, cardiovascular disease, respiratory disease (excluding asthma), obesity, and immunocompromise.

†† Influenza vaccination campaign start dates were Argentina: March 21; Brazil (except for the Northern Region): March 25, Chile: March 13, Paraguay: April 3, and Uruguay: April 24.

§§
https://www.who.int/publications/m/item/recommended-composition-of-influenza-virus-vaccines-for-use-in-the-2024-southern-hemisphere-influenza-season

¶¶ VE was estimated using multivariable logistic regression as (1 − adjusted odds ratio) × 100%.

*** 45 C.F.R. part 46.102(l)(2), 21 C.F.R. part 56; 42 U.S.C. Sect. 241(d); 5 U.S.C. Sect. 552a; 44 U.S.C. Sect. 3501 et seq.

†††
https://gisaid.org

§§§
https://joss.theoj.org/papers/10.21105/joss.03773

¶¶¶
https://www.cdc.gov/flu/prevent/prevention.htm

****
https://population.un.org/wpp
